# Evolution of Mass Movements near Epicentre of Wenchuan Earthquake, the First Eight Years

**DOI:** 10.1038/srep36154

**Published:** 2016-11-08

**Authors:** Shuai Zhang, Limin Zhang, Suzanne Lacasse, Farrokh Nadim

**Affiliations:** 1 Department of Civil and Environmental Engineering, The Hong Kong University of Science and Technology, Clear Water Bay, Hong Kong; 2 Faculty of Science and Technology, Technological and Higher Education Institute of Hong Kong, Tsing Yi, Hong Kong; 3 Norwegian Geotechnical Institute, Oslo, Norway

## Abstract

It is increasingly clear that landslides represent a major cause of economic costs and deaths in earthquakes in mountains. In the Wenchuan earthquake case, post-seismic cascading landslides continue to represent a major problem eight years on. Failure to anticipate the impact of cascading landslides could lead to unexpected losses of human lives and properties. Previous studies tended to focus on separate landslide processes, with little attention paid to the quantification of long-term evolution of multiple processes or the evolution of mass movements. The very active mass movements near the epicentre of the Wenchuan earthquake provided us a unique opportunity to understand the complex processes of the evolving cascading landslides after a strong earthquake. This study budgets the mass movements on the hillslopes and in the channels in the first eight years since the Wenchuan earthquake and verify a conservation in mass movements. A system illustrating the evolution and interactions of mass movement after a strong earthquake is proposed.

Large earthquakes induce widespread landslides that cause significant spatial erosion^1^,^2^,^3^,^4^,^5^. Elevated landsliding activities after a strong earthquake can continue to cause losses of life and property for years or even decades. A variety of surface processes exist by which materials can be moved over time through hillslope systems^6^. These processes are generically known as mass movements or mass wasting^7^. More than 60,000 landslides were triggered by the 12 May 2008 Wenchuan earthquake in China, with 25–30,000 fatalities caused by the earthquake-induced landslides^5^. The major hazards following the strong earthquake included large slides and rock falls, large-scale debris flows, landslide dams, flooding due to dam breaching, increased sedimentation, change of river course, and scouring. These hazards evolved either separately or as an integrated system known as cascading landslides. The mass movements after a mega-earthquake and their impacts should be considered over the full chain of the cascading landslide processes. Previous studies focused on single processes^3^,^5^,^8^,^9^,^10^ with little attention paid to multiple processes or evolutions of mass movements^11^. Such a limitation poses challenges to hazard mitigation and preparedness: how to evaluate quantitatively the long-term impact of a strong earthquake on the mass movements? How does the volume of erodible sediment residual to earthquake- and rain-induced mass movements change over time? How do the multiple hazards transform and interact in the evolution process? This paper examines the evolution of the long-term mass movement after the 2008 Wenchuan earthquake in space and in time. The study reveals a conservation in mass movements in the first eight years since the Wenchuan earthquake. A system illustrating the evolution and interactions of mass movement after a strong earthquake is proposed.

## Mass movement events after the Wenchuan earthquake

**Figure 1:**
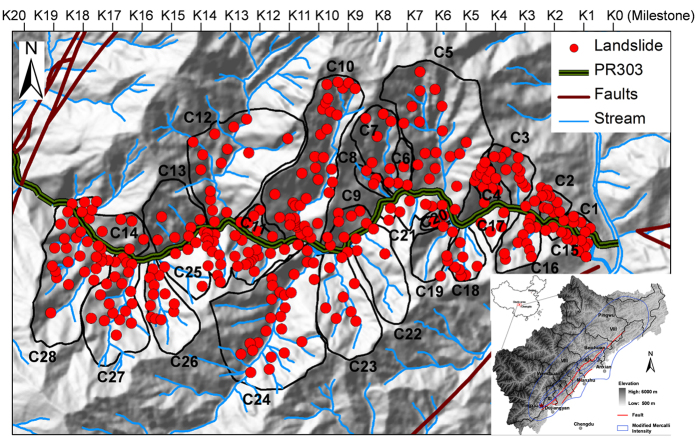
Locations of landslides along highway PR303 shortly after the Wenchuan earthquake. The study area is located near the epicentre, Yingxiu. The figure was generated using ArcGIS, version 9.3.1, http://webhelp.esri.com/arcgisdesktop/9.3/index.cfm?TopicName=welcome.

**Figure 2:**
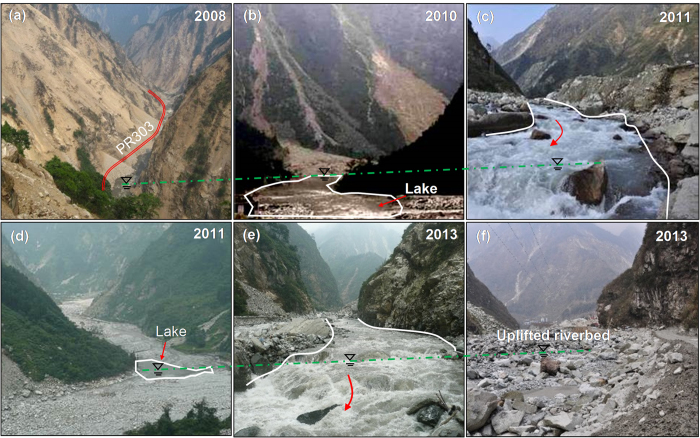
Evolution of the topography during 2008–2013: (**a**) landslides triggered by the earthquake (at K5.5 as indicated in Fig. 1); (**b**) debris flows induced by the rainstorm on 13 August 2010; (**c**) dam breach in 2011; (**d**) uplifted riverbed after the rainstorm on 3 July 2011; (**e**) dam breach in 2013; (**f**) uplifted riverbed after the rainstorm on 10 July 2013 (photos in b, c, d, e, f were taken at K6 as indicated in Fig. 1, near the gully mouth of catchment C5).

Extreme mass movements occur frequently following a strong earthquake^12^,^13^,^14^,^15^,^16^,^17^. The epicentre of the Wenchuan earthquake, Yingxiu, was very close to the Province Road PR303 (PR303) leading to the Research and Conservation Centre of Giant Pandas at Wolong. A total of 28 catchments with a combined area of 85 km^2^ along highway PR303 were selected for this study (Fig. 1). During the earthquake in 2008 and in the eight years after the earthquake, PR303 experienced multiple extraordinary hazards (Fig. 2). Widespread co-seismic landslides were triggered on both sides of the road during the earthquake. The landslides cut off the roads and isolated the stricken region, causing numerous fatalities (Fig. 2a). The reconstruction of PR303 started in April 2009. Although efforts have been made to remove or strengthen some unsafe deposits, many deposits at high elevations are still unstable.

During the rainy season, a large amount of landslide materials loosened during the earthquake evolved into debris flows along PR303 on 14 August 2010, 3 July 2011 (Fig. 2b,d) and 10 July 2013. Some landslides blocked a major river (the Yuzixi River), forming landslide dams. The collapse of the landslide dams caused floods and cut off the highway repeatedly (Fig. 2c,e). The riverbed along the road rose at least 30 m due to the accumulation of sediments during the past eight years (Fig. 2f). These cascading hazards not only caused losses of life and property, but also initiated a fundamental transformation of the natural environment. Such transformation is expected to last for many years. Understanding sediment generation, transport, and rate of mass movement has significant implications for global environmental and social sustainability and risk management. Compared with descriptive studies at other sites in the Wenchuan earthquake zone^17^, the mass movements in the selected study area were monitored continuously in the past eight years.

## Results and Discussion

### Volumetric balance in mass movement

**Figure 3:**
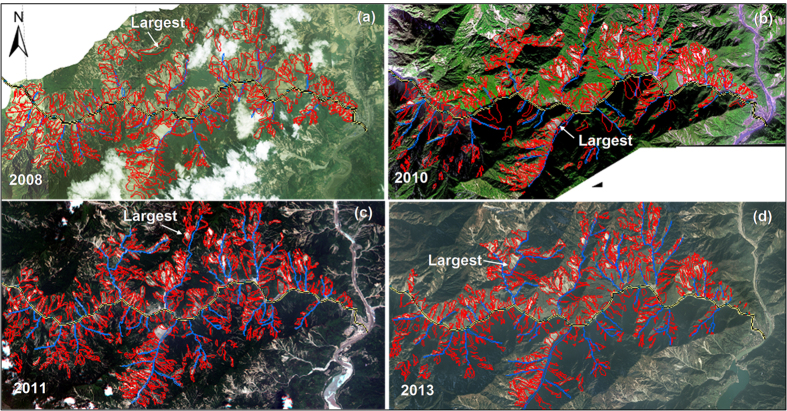
Satellite images after the earthquake and inventories of landslides: (**a**) landslides triggered by the earthquake (image from Quickbird satellite, which was taken on 30 May 2008 with a resolution of 0.61 m); (**b**) fresh landslides induced by the rainstorm on 13 August 2010 (image from Worldwide-2 satellite, which was taken on 18 December 2010 with a resolution of 2.5 m); (**c**) fresh landslides induced by the rainstorm on 3 July 2011 (image from RapidEye satellite, which was taken on 8 July 2011 with a resolution of 5 m); (**d**) fresh landslides induced by the rainstorm on 10 July 2013 (image from SPOT-6 satellite, which was taken on 1 December 2013 with a resolution of 1.5 m). The fresh landslides included reactivated shallow landslides on the pre-existing landslides from earlier events, and new landslides occurring at locations not affected by earlier events.

**Figure 4:**
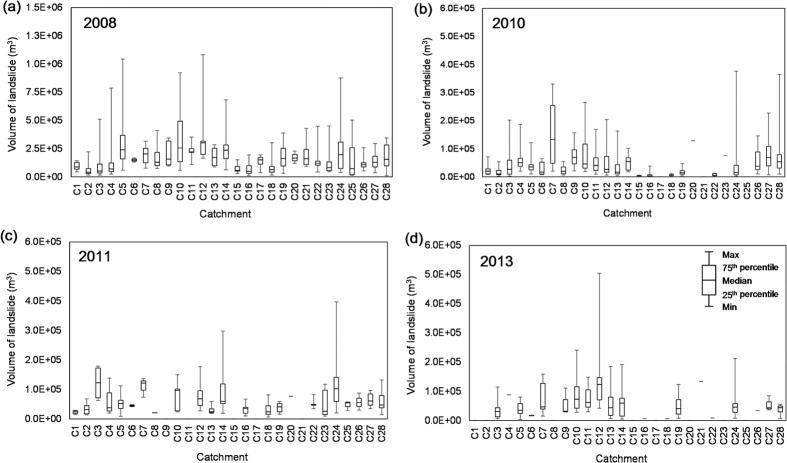
Volumes of fresh landslides distributed along the highway, occurring in the events of (**a**) May 2008; (**b**) August 2010; (**c**) July 2011; (**d**) July 2013.

In order to quantify the significant impact of the Wenchuan earthquake on the subsequent mass movements, four landslide inventories in the study area (Fig. 3) were generated, based on satellite images taken in 2008, 2010, 2011 and 2013, and more than ten rounds of comprehensive field investigations. In 2008, the dominant hazard was earthquake-induced landslides; in the subsequent years from 2010 to 2013, debris flows became the dominant hazard. We examined the covering areas of the landslides delineated on an ArcGIS platform. The volume of each landslide in the four inventories was quantified by multiplying the area covered by the landslide by the estimated average thickness of the deposit (Fig. 4).

Uncertainties exist in evaluating the landslide covering area and the thickness of the deposits. The uncertainties in area measurements range from 0.6% to 5% for a deposit 200 m × 200 m in size since the area is measured based on satellite images with varying resolutions of 0.61 m, 2.5 m, 5 m, and 1.5 m in 2008, 2010, 2011 and 2013, respectively. The thickness of soil deposits is the most difficult to estimate because it is not observable remotely. Twenty-five selected hillslope deposits were investigated in detail during our field investigations conducted in March 2009, December 2010, December 2011 and December 2013. Based on the statistical analysis of the deposit thicknesses in the scar area and the deposition area, the coefficient of variation (COV) of the deposit thickness ranges from 5.5% to 15% for the scar area, and from 12.4% to 17.6% for the deposition area in the evaluation years of 2008, 2010, 2011 and 2013. The uncertainties in thickness measurements are mainly caused by unexpected variations of terrain geometry. Finally, the uncertainties in volume estimation range from 13.3% to 14.5% (Table 1).

The box plots in Fig. 4 display the full range of variations of the landslide volume (from the minimum to the maximum) for the 28 catchments of various watershed areas along the highway (Fig. 1). The box spans the inter-quartile range, the line within the box denotes the median and the whiskers denote the 25^th^ and 75^th^ percentiles, respectively. Each catchment has significantly different medians.

#### 2008

In 2008, a total of 305 hillslope deposits and 28 channel sediment deposits triggered by the earthquake were identified in the 85 km^2^ study area^16^ (Fig. 3a). Here a hillslope deposit refers to a deposit of loose material of a landslide triggered by an earthquake or subsequent rainfall with a large slope angle. The mean slope angle of the hillslope deposit can be affected by climate and failure frequency^18^. A channel sediment deposit is accumulation of loose materials in a channel, which is typically deposited, eroded, and redeposited repeatedly in a stream channel, especially during climatic variations. The total volume of the 305 hillslope deposits was 57.5 × 10^6^ m^3^ and the total volume of the 28 channel sediment deposits was 12.4 × 10^6^ m^3^ (Table 2). The slope gradients of these loose deposits ranged from 6° to 48°. The largest landslide was No. 113 in catchment C12 with a slope gradient of 21°, covering an area of 0.46 km^2^ and a volume of 1.08 × 10^6^ m^3^ (Fig. 3a), located at elevations between 2300 m and 3190 m^16^.

#### 2010

The 13 August 2010 storm was the first most severe rainstorm occurring since the earthquake. After this storm, a total of 590 hillslope loose deposits with a volume of 48.9 × 10^6^ m^3^ were identified: 351 were fresh landslides induced by the storm (Fig. 3b) and the remaining 239 were the stable deposits of landslides induced by the earthquake in 2008. The 351 fresh landslides included 322 reactivated shallow landslides on the existing landslide deposits triggered in 2008, and 29 new landslides which occurred at locations that had not failed in 2008. The largest one was located in catchment C24, covering an area of 0.16 km^2^ with a volume of 0.38 × 10^6 ^m^3^ (Fig. 3b). The number of channel sediment deposits increased to 35 with a total volume of 16.9 × 10^6 ^m^3^ after the storm^16^.

#### 2011 and 2013

During the rainstorm in July 2011, 157 fresh landslides were induced and 507 hillslope deposits were recognized (Fig. 3c). These fresh landslides included 133 reactivated shallow landslides and 24 new landslides at locations that had not failed in 2008 or 2010. The total volume of the 507 hillslope deposits was 44.1 × 10^6 ^m^3^. Furthermore, the number of channel sediment deposits was 43 and the materials in the channels increased to 19.2 × 10^6 ^m^3^ (Table 2). In 2013, the number of fresh rain-induced landslides was 135 (Fig. 3d). A total of 50 channel sediment deposits were identified. The total volumes of the hillslope deposits and channel sediment deposits were 40.7 × 10^6 ^m^3^ and 20.4 × 10^6 ^m^3^, respectively (Table 2).

#### Evolution of areas affected by the landslides

Based on aerial photographs shortly after the earthquake in 2008, the total area of the earthquake-triggered landslides in 2008 was 24.02 km^2^ and the earthquake-induced landslide ratio (definition in the last section) was 28.25%. The landslide covering area decreased over time (Table 2). After the rainstorm in 2013, the area of all types of landslides decreased to 18.31 km^2^. The rain-induced fresh landslide area (including the areas of both reactivated and new landslides) was 8.79 km^2^, corresponding to a landslide ratio of 10.3%, which is only one third of the earthquake-induced landslide area in 2008. The reactivation ratio (definition in the last section) of earlier landslides was 49% for the rainstrom in 2010, 25% in 2011, and 22% in 2013. The ratio of new landslides at locations that had not failed earlier (or since 2008) was 9% due to the storms in 2010, 14% in 2011, and 17% in 2013. However, the total number of landslide deposits changed from 305 in 2008 to 625 in 2013 (Table 2).

What caused the changes in the extent and density of rain-induced landslides? During the few years after the earthquake, the vegetation gradually recovered, thus alleviating the erosion of the loose materials during the rainy season. A rainstorm often caused an original loose deposit to lose stability, but only locally. A single original debris deposit could develop several small local failures, and the dominant type of landslides transformed from debris slides to debris flows^16^. This is one reason for the increasing number of landslide deposits but decreasing total landslide area.

#### Volume balance

**Figure 5:**
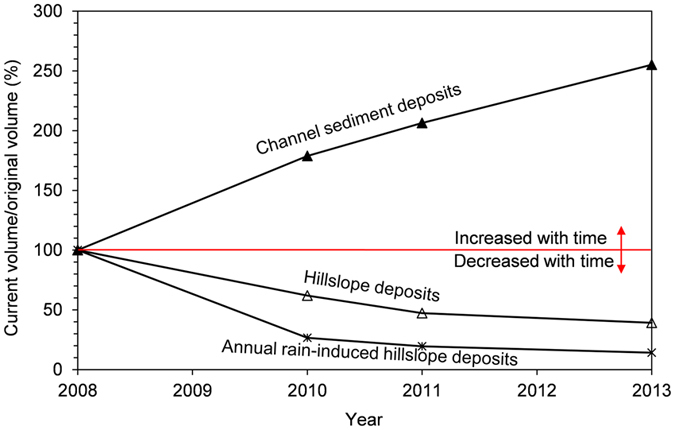
Change in volume with time of loose soil deposits in active catchments in the 85 km^2^ study area. “Channel sediment deposits”, “hillslope deposits”, and “annual rain-induced hillslope deposits” refer to those deposits distributed in the active catchments in which debris flows occurred during 2008–2013. The annual rain-induced hillslope deposits include the reactivated landslides which occurred on the pre-existing landslides induced before, and the newly triggered landslides. The hillslope deposits include the reactivation landslides, the newly triggered landslides and the relict of the original landslides.

**Figure 6:**
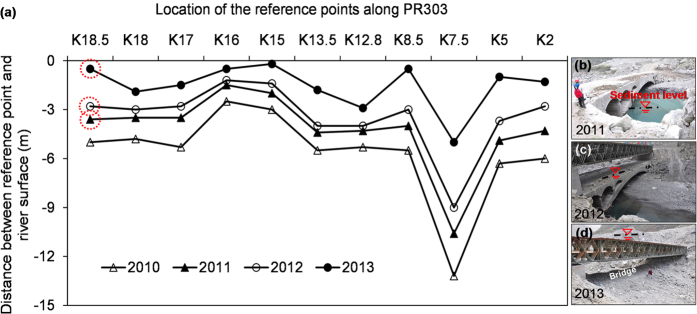
(**a**) Evolution of the riverbed after the Wenchuan earthquake; (**b**) aggraded riverbed at reference point K18.5 (photo taken in Dec. 2011); (**c**) aggraded riverbed in Dec. 2012; (**d**) aggraded riverbed in Dec. 2013 (The locations of the river reference points are shown in Fig. 1).

During each storm event in the period of 2008–2013, some of the hillslope deposits evolved into channel sediment deposits and the materials in the channels gradually moved towards the gully mouth. Thus the volume of the hillslope loose deposits decreased while the volume of the channel sediment deposits increased (Fig. 5), especially for two active catchments (e.g. C06 and C07) where three repeated debris flows occurred during the interval 2008–2013 (Fig. 5). The rates of mass transport, i.e. the percentage volume loss of the hillslope deposits, for the active debris flow catchments, are respectively 24.5%, 16.3% and 12.1% due to the storm events in 2010, 2011 and 2013, showing an obvious decreasing trend (Fig. 5). Meanwhile the volume of the channel sediment deposits in the active catchments increased dramatically (Fig. 5). The solid materials triggered by the earthquake were rearranged in space during the past eight years. The total amount of mass movement in each year is estimated by summing up the runout materials of the debris flows occurring in that year, the hillslope loose deposits, and the channel sediment deposits retained in the catchments. The runout materials refer to the debris flow materials that ran out of the ravine mouth and deposited on a debris flow fan. By examining the balance of the volumes of the hillslope deposits, channel sediment deposits, and runout materials during the three storm events, it is found that the total amount of mass during a landslide process is approximately “conserved” over time, i.e. ranging from 69.9 × 10^6 ^m^3^ in 2008 to 62.3 × 10^6 ^m^3^ in 2013 (Table 2). The reduction in the volume of loose materials is due to the washing away of materials through sediment transportation and the uncertainties in the volume estimation, which together with part of the runout materials contributed to the aggraded riverbed (Fig. 6). Excluding the loss of suspended sediments with water flow and the loss of materials due to manual dredging, the quantities of sediment contributing to the aggraded riverbed were approximately 1.2 × 10^6 ^m^3^ in year 2010, 0.7 × 10^6 ^m^3^ in year 2011, and 1.5 × 10^6 ^m^3^ in year 2013, respectively. Approximately 90% of the erodible material still remains on the hillslope and channels. These findings imply that the landslide material has transformed to different forms over time, but that the total mass remains constant.

### Multiple landslide hazards – separate evolution

#### Slope failures

The catastrophic Wenchuan earthquake not only triggered a large number of co-seismic landslides but also seriously disturbed the surficial strata. Some substrata on the steep hill slopes became unstable due to the presence of numerous tension cracks induced by the strong ground motion. Rock falls often occurred when detached materials fell down from high elevations. These factors undoubtedly intensified landslide activities.

Besides, reactivated landslides which occurred on the pre-existing landslides were triggered by intense rainfall, water infiltration and decrease in the shear strength of the soil^12^,^19^. Under normal weather conditions, the hillslope deposits along PR303 are in a quasi-stable state. These deposits may become unstable and reactivate when subjected to intense precipitation.

#### Debris flows

After suffering several extreme rainstorms in 2010, 2011, and 2013, a large quantity of shallow landslide materials “primed” by the earthquake rapidly evolved into debris flows, which moved along pre-existing channels onto deposition fans or into rivers. With the movements of the solid materials, the source materials for the debris flows evolved gradually, the hillslope deposits developing into channel sediment deposits and the solid materials in the channels moving downward. As the process continued, the hillslope debris flows became less frequent while the channelized debris flows that initiated due to channel-bed failure gradually became more dominant^20^. With the occurrence of repeated debris flows, the debris fan materials became coarser over time. The debris flow fronts in the 2008 event contained a substantial fraction of fines, while the debris flow fronts in the 2013 event contained primarily coarse particles^20^. Nearly all the debris flows were characterized by increasingly coarse boulder fronts. After these debris flows, a large amount of loose materials still remained in the catchments (Fig. 5), forming potential source materials for new debris flows in the future.

#### Landslide dams, dam-breach floods and elevated riverbed

During and after the 2008 earthquake, the runout materials of the earthquake-induced landslides blocked the Yuzixi River and formed landslide dams in numerous places. Most of these landslide dams were breached in the rainy season shortly after the earthquake in 2008, and the resulting sediments redistributed along the river, raising the riverbed by at least 10–16 m compared with that before the earthquake. During the rainy season in August 2010, a large amount of debris material was brought into the river by the newly developed debris flows. The landslide dams formed in 2010 were breached during the 4 July 2011 rainstorm, and the continued sedimentation caused an additional 1 to 3 m of riverbed rise along the river (Fig. 6). During the debris flow event in 2013, more landslide lakes formed and were overtopped. The redistributed sediments contributed to further aggradation of the riverbed, bringing the total riverbed rise to 12–25 m along the river. As Figs 6b–d show, a bridge disappeared due to the elevated riverbed. In these figures, the landslides contributed to the aggradation of the riverbed during the past eight years.

### Landslide mass movement chain

**Figure 7:**
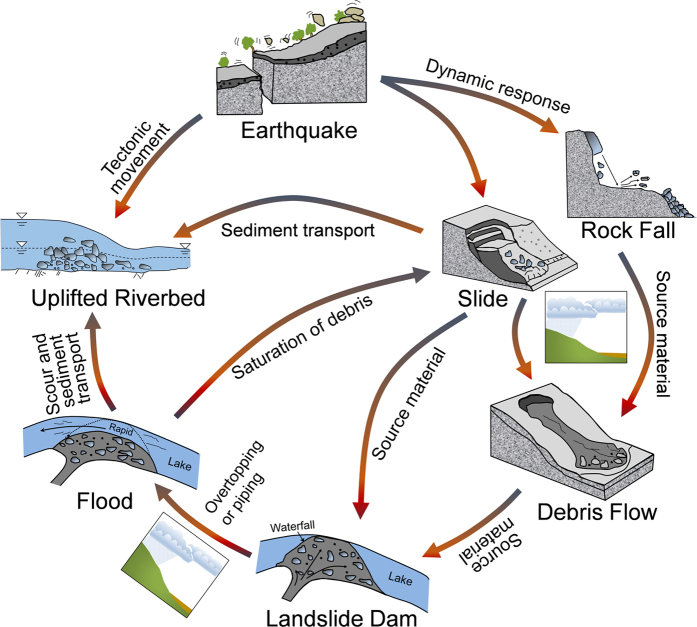
Hazard-response chain of mass movement processes following a strong earthquake: the periphery gives the six natural geo-phenomena; the inside arrows illustrate the processes and transition leading to increased hazards due to an initial strong earthquake trigger.

The mass movements after the Wenchuan earthquake can be expressed by a hazard-response chain, i.e. a process from loose materials from the earthquake-induced landslides to the aggraded riverbed, and from a disturbed state to a new state after the system reaches a balance. Figure 7 presents a hazard-response chain of mass movement processes following a strong earthquake, based on the experience from the events following the 2008 Wenchuan earthquake. Six leading natural phenomena (i.e. landslide, rock fall, debris flow, landslide dam, flood and aggraded riverbed) are included in the chain and form the outer periphery. The arrows within the figure illustrate the processes and transition that lead to increased hazards after an initial strong earthquake.

A strong earthquake can trigger slides and rock falls, either simultaneously or consecutively. Under heavy rainfall, the colluvium materials spreading out on steep hill slopes can reactivate, move downwards and become channel sediment deposits. The materials in the channels can then gradually run out as channelized debris flows under the same or a subsequent rainstorm. In some cases, the materials from a hillslope slide, a rock fall or a debris flow can block a river and form a landslide lake. Flooding due to overtopping occurs if the dam breaches or fails due to piping. The debris transported by the flood elevates the riverbed. A flood and erosion at the toe of a hillslope can also prompt additional slope failures as the soil materials become saturated or scoured by the flood water. The evolution will not stop before the entire system reaches a new balance.

The dominant hazard evolves over time. The transformation and interrelationships among the hazards follow a network scheme, as illustrated in Fig. 7. Shortly after the Wenchuan earthquake, slides and rock falls were dominant. With the hillslope deposits evolving into channel sediment deposits, the rain-induced debris flows became dominant in the period 2 to 5 years after the earthquake. As the repeated debris flows occurred, the fine particles in the soils eroded due to surface flow. Over time more large particles became exposed, leading to an increased triggering threshold and decreased debris flow activity. However, at present the debris flow hazards are still in an active stage in which many debris flows can outbreak in a large storm as a tremendous amount of loose material is suspended on the hill slopes, ready to be eroded and transported. The flood hazards may gradually become dominant. The landform today reflects the changes due to the many factors that have occurred over time. Processes that shape the earth surface are on-going, continuously or intermittently. The hazard-response chain in Fig. 7 describes the dynamic transitions among the six mass movement processes (landslide, rock fall, debris flow, landslide dam, flood and aggraded riverbed), and explains the interrelationships and interactions among the six geo-phenomena, and how the relative importance and preponderance of each can change over time.

## Conclusions

The primary hazards following a strong earthquake include earthquake-induced slides and rock falls, and subsequent rain-induced slides, debris flows, floods due to landslide dam breaching and aggraded riverbeds. These hazards may either evolve separately or simultaneously. The authors investigated the landslide mass movements near the epicentre of the 2008 Wenchuan earthquake in the first eight years after the earthquake. Four inventories of landslides in 28 catchments along a major highway near the epicentre of the earthquake were prepared for the investigation. The volume of the hillslope loose deposits decreased while the volume of the channel sediment deposits increased over time. The mass transport rates were 24.5%, 16.3% and 12.1% during the rainstorms in 2010, 2011 and 2013, respectively. The quantities of the sediment that contributed to the aggraded riverbed were approximately 1.2 × 10^6 ^m^3^ in year 2010, 0.7 × 10^6 ^m^3^ in year 2011, and 1.5 × 10^6 ^m^3^ in year 2013, respectively. Approximately 90% of the erodible material still remains on the hillslope and channels. Such mass movement process indicates that the mass is redistributed with time or rearranged in space. The eight-year records of mass movements also demonstrate that the riverbed aggradation in the study area is mainly related to landslide-related events.

Redistribution of materials contributed to the evolution of dominant hazards in different periods. The authors propose a hazard-response chain to describe the mass movement process following a strong earthquake, based on the experience from the events that followed the 2008 Wenchuan earthquake. Six lead natural mass movement processes (i.e. landslide, rock fall, debris flow, landslide dam, flood and aggraded riverbed) are included. The hazard-response chain explains how the six hazards relate to each other and how the process changes from one type of hazard to another over time. The association of the aggraded riverbed with the successive landsliding events implies a dynamic process of the hazards and the mass movements after a strong earthquake.

## Methods

### Landslide mapping

Digital photo interpretation techniques and field verifications were combined to characterize the hillslope deposits, channel sediment deposits and runout materials in the study area. Quickbird images taken on 30 May 2008 (shortly after the Wenchuan earthquake), Worldwide-2 images taken on 18 December 2010 (shortly after the August 2010 debris flows), RapidEye images taken on 8 July 2011 (shortly after the July 2011 debris flows), SPOT-6 images taken on 1 December 2013 (after the July 2013 debris flows), satellite images from Google Earth Pro taken on 15 April 2015 were prepared. The resolutions of the Quickbird, Worldwide-2, RapidEype, SPOT-6, and Google Earth Pro images were 0.61 m, 2.5 m, 5 m, 1.5 m and 0.5 m, respectively. These images provide data on the areas of landslide and the areas of disturbed ground likely to be associated with debris flows. Important aspects in the recognition of landslides are the size of the landslide features, the difference in spectral characteristics between the landslides and their surrounding areas, and the morphological expression^21^. Features such as scarps, disrupted vegetation cover, and the state of landslide deposits were used in conjunction with the morphological features. In the recognition of debris flows, the boundaries of the catchments, the source areas, the transportation channels and the deposition zones of debris flows can be directly delineated using these satellite images with the assistance of an ArcGIS platform. The satellite images in 2015 were not interpreted since no extreme storm occurred in 2015. A digital elevation model (DEM) derived from digitized contours at 20 m intervals, mapped by Sichuan Highway Department in December 2008, was used to study the topography, geology and channel system in the study area. The slope geometry was described using the DEM on ArcGIS. More than ten rounds of field investigations were conducted at the site from March 2009 to December 2015 to validate the identification of hillslope and channel sediment deposits, examine the mass movement paths and erosion features, survey the depositional fans, measure the volumes and grain sizes of the run-out materials, and record the uplifted elevation of the riverbed.

### Volume estimation

The volume of the run-out debris flow material, the hillslope deposits and the sediment in the river can be estimated by multiplying the landslide area by the average thickness of the deposits in each scenario^22^. The thickness of the run-out debris was determined at selected locations by borehole drilling in the middle part of the debris fans, trenching in the frontal areas and direct measurement at exposed locations around the debris fans. The deposition areas of the debris flows and the areas of the hillslope deposits were determined on an ArcGIS platform based on the satellite images. By measuring the scar and deposition areas of 25 hillslope deposits and channel sediment deposits accessible during our field investigations, the average ratio of the scar area to the deposition area was determined as 1:3. The deposit thicknesses in the scar area and the deposition area, measured using a laser range finder *in-situ*, were on average 1.07 m and 3.08 m in 2008; 1.03 m and 3.03 m in 2010; 0.98 m and 2.95 m in 2011, and 0.95 m and 2.8 m in 2013, respectively. The average thickness of the channel sediment deposits was 10 m. The volume of each loose soil deposit or channel sediment deposit can then be evaluated. The materials from many shallow landslides spread in large areas on the steep terrain in the study area, which differ from the sample landslides employed to develop the area-volume scaling relationships^3^,^5^. Therefore these relationships are not used in this paper.

Assuming the cross section of the river channel to be rectangular as the river banks are rather steep, the total volume of the riverbed sediments can be evaluated by multiplying the covering area of the river channel by the average riverbed rise in each scenario. The surface area of the riverbed sediment was obtained based on the interpretation of satellite images. The riverbed rise was obtained by measuring the distance between the reference point and the riverbed surface after each hazard event; namely in December 2010, 2011 and 2013 (Fig. 6).

### Landslide ratio

A few landslide ratio terms are used to quantify the mass movements within the study area in different periods. The earthquake-induced landslide ratio is defined as the area of earthquake-induced landslides (24.02 km^2^) divided by the total study area (85 km^2^). The reactivated ratio is the ratio of the reactivated landslide area to the old landslide area prior to each storm event. The mass movement rate is the sum of the increased channel sediment deposits and runout materials identified in current year, divided by the amount of the hillslope deposits in the previous year.

## Additional Information

**How to cite this article**: Zhang, S. *et al*. Evolution of Mass Movements near Epicentre of Wenchuan Earthquake, the First Eight Years. *Sci. Rep.* **6**, 36154; doi: 10.1038/srep36154 (2016).

**Publisher’s note:** Springer Nature remains neutral with regard to jurisdictional claims in published maps and institutional affiliations.

## Figures and Tables

**Table 1:** Uncertainties in estimating the volume of loose deposits from 2008 to 2013.

Year	COV* of area (%)	COV of thickness (%)		COV of volume (%)
		Scar area	Deposition area	
2008	0.6	15.0	12.4	13.3
2010	2.5	9.8	15.7	13.7
2011	5.0	5.5	17.6	13.5
2013	1.5	10.6	17.1	14.9

^*^Coefficient of variation.

**Table 2:** Volume balance from 2008 to 2013.

Type	Hillslope deposit*				Channel sediment deposit				Runout material			Total			
Year	2008	2010	2011	2013	2008	2010	2011	2013	2010	2011	2013	2008	2010	2011	2013
Number of loose deposits	305	590	507	625	28	35	43	52	26	17	21	—	—	—	—
Total area (km^2^)	24.0	20.7	19.2	18.3	1.2	1.7	1.9	2.0	2.7	1.6	1.9	25.3	25.0	22.7	22.2
Volume (10^6 ^m^3^)	57.5	48.9	44.1	40.7	12.4	16.9	19.2	20.4	3.1	0.9	1.2	69.9	68.9	64.2	62.3

^*^Hillslope deposits include reactivation landslide deposits, newly occurring landslide deposits, and stable deposits of the landslides induced by prior events.
